# Anti-NMDA-Receptor GluN1 Antibody Serostatus Is Robust in Acute Severe Stroke

**DOI:** 10.3390/diagnostics15243132

**Published:** 2025-12-09

**Authors:** Pia Sophie Sperber, Benjamin Hotter, Matthias Endres, Harald Prüss, Andreas Meisel

**Affiliations:** 1 Department of Neurology and Experimental Neurology, Charité-Universitätsmedizin Berlin, 10117 Berlin, Germany; 2 Experimental and Clinical Research Center, 13125 Berlin, Germany; 3 Neuroscience Clinical Research Center, Charité-Universitätsmedizin Berlin, 10117 Berlin, Germany; 4 Germany Center for Cardiovascular Diseases (DZHK), 10117 Berlin, Germany; 5 Center for Stroke Research Berlin, Charité-Universitätsmedizin Berlin, 10117 Berlin, Germany; 6 German Center for Neurodegenerative Diseases (DZNE), 10117 Berlin, Germany

**Keywords:** stroke, antibodies, NMDA-receptor, biomarker

## Abstract

**Background:** Anti-N-methyl-D-aspartate IgM and IgA antibodies (NMDAR1-abs) are associated with unfavorable stroke outcomes and may be risk factors thereof. However, to utilize NMDAR1-abs serostatus for risk assessment in acute stroke, it is crucial to understand the robustness of serostatus during this phase. Therefore, we investigated the robustness of NMDAR1-abs serostatus and titer levels up to seven days after stroke. **Methods:** In this exploratory analysis of the multicenter STRAWINSKI trial (identifier: NCT01264549), patients with severe ischemic stroke (NIHSS ≥ 9) in the middle cerebral artery territory were included. The first blood sample was taken within 36 h and then daily from day two to seven after stroke. NMDAR1-abs immunoglobulin (Ig)A and IgM were assessed in serum using cell-based assays. We initially measured NMDAR1-abs in the total cohort on day 1. Subsequently, in samples from seropositive and matched seronegative patients, we measured NMDAR1-abs on each following day. Titer dilutions started from 1:10 up to 1:1000. Seropositivity was defined as any titer > 0. **Results:** Out of 171 patients (mean age = 76 [SD = 11], median NIHSS = 15 [IQR = 12–18]), 16 (9%) individuals were seropositive. Seropositive patients remained seropositive and matched seronegative participants remained seronegative over sequential measurements. Although titer levels remained largely unchanged, some patients showed fluctuating titers. **Conclusions:** The status of NMDAR1-abs seropositivity is stable during acute stroke, with little to no variation in titer levels.

## 1. Introduction

Anti-N-methyl-D-aspartate antibodies (NMDAR1-abs) were first described in the context of autoimmune encephalitis [1]. A high prevalence of NMDAR1-abs of the immunoglobulin (Ig)M and IgA isotypes was found in people with various diseases including, presumably, healthy individuals and stroke patients [2,3,4].

In recent years, growing efforts have aimed to elucidate the role of NMDAR1 antibody (NMDAR1-abs) seropositivity in the context of stroke [5,6]. Multiple observational studies across diverse populations have demonstrated associations between NMDAR1-abs and outcomes [6,7]. In stroke, seropositivity has been linked to an increased risk of secondary vascular events and unfavorable long-term cognitive outcomes [8,9,10]. Additionally, high titers of these antibodies were linked to worse functional outcome [8]. These findings suggest a potential relevance of NMDAR1-abs in stroke, either as a therapeutic target or as diagnostic tool. However, the clinical utility of biomarkers largely depends on the reliability and stability of their measurement. To date, most studies have relied on a single NMDAR1-abs measurement during the acute phase of stroke, and additionally patients were often only mildly affected. Thus, it remains unclear whether seropositivity is influenced by the timing of measurement post-stroke or the severity of the stroke event.

In this study, we leveraged data and biospecimens from a rigorously conducted multicenter trial to address three critical questions regarding the potential use of NMDAR1-abs as a biomarker in acute stroke. Specifically, we sought to determine the following: (1) the prevalence of NMDAR1-abs seropositivity in patients with severe stroke; (2) whether NMDAR1-abs seropositivity is a robust marker during the acute stroke phase; and (3) whether NMDAR1-abs titer levels remain stable across consecutive measurements.

## 2. Material and Methods

### 2.1. Study Design and Patient Population

We used serum samples and data from the ‘Stroke Adverse Outcome Is Associated with Nosocomial Infections: PCTus-Guided Antibacterial Therapy in Severe Ischemic Stroke Patients (STRAWINSKI)’ study (ClinicalTrials.gov NCT01264549), a randomized controlled trial investigating the role of early procalcitonin measurement as guidance for antibiotic treatment. Details on the study design were published previously [11,12]. Briefly, patients of at least 18 years of age, with a severe stroke event, defined by the National Institute of Health Stroke Scale (NIHSS) > 9 in the middle cerebral artery (MCA) territory within the past 36 h were included at ten study centers in Berlin and Brandenburg, Germany. Patients with hemorrhage in computer tomography or magnet resonance imaging (MRI), modified Rankin Scale (mRS) on admission ≥ 4, antibiotic treatment within the past 10 days, life-expectancy less than three months, pregnancy or lactation were excluded [11].

### 2.2. Blood Sampling and Anti-NMDA-Receptor Antibody Measurement

Sera were collected upon study enrollment and then daily from the second to the seventh day after the stroke following a strict protocol. NMDAR1-abs of the immunoglobulin (Ig)A and IgM were measured by two independent raters with cell-based assays using commercially available test kits (EUROIMMUN) in an accredited laboratory. Titers were secondary, confirmed by a senior laboratory physician. Assessors were unaware of any patient information, including which sera belonged to whom at which specific time-point. Titer levels ranged from 1:10 to 1:1000 and any titer from 1:10 was considered seropositive. Measurements were conducted in two batches: first, all available samples from baseline visits; second, seropositive individuals were matched to seronegative individuals, and we examined NMDAR1-abs serostatus in this subset for up to 6 consecutive days following the baseline visit.

### 2.3. Definition of Titer Change

We considered (i.) a change from NMDAR1-abs seropositive to seronegative status (or vice versa) or else, (ii.) a change of more than one titer dilution step, as a relevant titer change. The first (initial) measurement was compared to the last available measurement.

### 2.4. Clinical Outcome Exploration

Outcome data were available from the post-acute stroke phase (i.e., 90 days after stroke) and after 6 months. We explored counts of death and unfavorable mRS with regard to NMDAR1-abs serostatus at both of these time-points. An unacceptable mRS was considered an mRS > 4.

### 2.5. Statistical Analyses

Based on the baseline measurement, we matched a same-size (1:1) seronegative control group by propensity scores, built from the variables of age, sex, previous stroke (yes/no), arterial hypertension before stroke (yes/no), and atrial fibrillation before stroke (yes/no), because we considered these variables to potentially impact the stroke (which led to inclusion) and NMDAR1-abs serostatus. We then matched 1:1 with k-nearest neighbor, setting the caliper at 0.25. We explored clinical outcomes with regard to NMDAR1-abs using counts and proportions at FU time-points. All statistical calculations and figure plots were conducted in R version 4.2.3 (15 March 2023) using the tidyverse and ggplot2 packages.

### 2.6. Ethics Statement

All participants included in this study or their legal guardian gave written informed consent for study participation [11]. The study was approved by the Ethics Committee of Charité–Universitätsmedizin Berlin on 16 December 2010 and followed guidelines framed by the declaration of Helsinki.

### 2.7. Data Availability

All data and analyses scripts used in this study are available from the corresponding author upon reasonable request.

## 3. Results

### 3.1. Baseline Data

From initially 235 individuals included in the study, 171 baseline sera were available for antibody measurement. Out of 171 patients, 16 (9%) were seropositive for NMDAR1-abs, 13 with IgM, and four with IgA isotype antibodies. From five of these individuals (all seronegative) clinical characteristics were missing. In 166 individuals with antibody measurement, the mean age was 76 (SD = 11), 95 (55%) were female, and the median NIHSS was 15 (IQR = 12–18). Seropositive patients tended to be male (69% vs. 42% in seronegative patients), all of them had a history of hypertension, and half of the cases had a diabetes mellitus diagnosis (50% vs. 27% in seronegative patients). One third of seropositive patients were living in a facility care already before the stroke. For an overview of all assessed characteristics, see Table 1. We were able to match 15 seronegative patients to seropositive patients according to the baseline assessment, rendering 31 individuals with whom we conducted follow-up measurements.

### 3.2. Follow-Up Data

**Figure 1:**
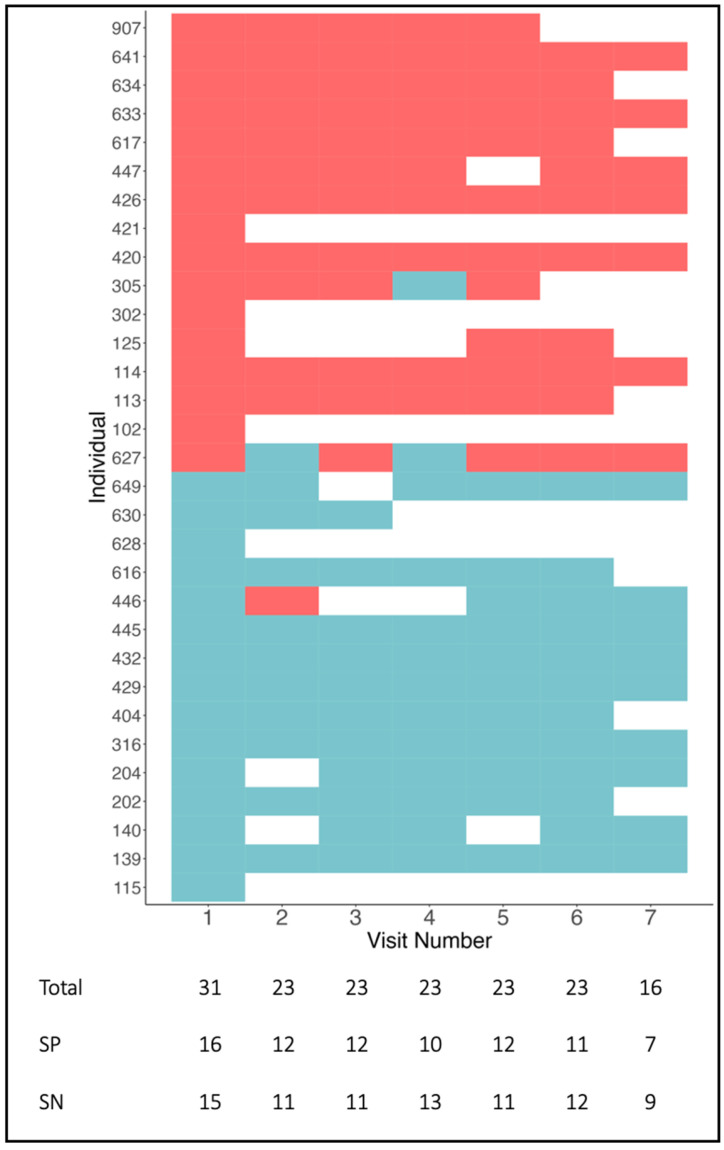
Heatmap displaying all NMDAR1-abs measurement results as seropositive (SP/red) and seronegative (SN/blue) for each sera ordered by individual and serostatus on day 1 (*y*-axis) over consecutive follow-up days (*x*-axis). One box represents the result of one sample, respectively.

Out of 16 seropositive patients and 15 matched seronegative patients (*n* = 31), we had at least one follow-up measurement in 26 individuals, who contributed a total of 131 sera for consecutive antibody measurement. From six patients of whom serum from follow-up visits was not available or too little, three were seropositive (1. IgM = 1:320; 2. IgM = 1:100; 3. IgA = 1:1000). Over follow-up measurements, seropositive patients remained seropositive, and seronegative patients remained seronegative (see Figure 1). Two seropositive patients were intermittently seronegative. One initially seronegative patient (i.e., for both Ig-isotypes) became seropositive with a low IgM titer of 1:10 at day two after the stroke, who, however, converted back to seronegative status on the next measurement. In total, out of 131 measurements, there were three conversions of serostatus.

**Figure 2:**
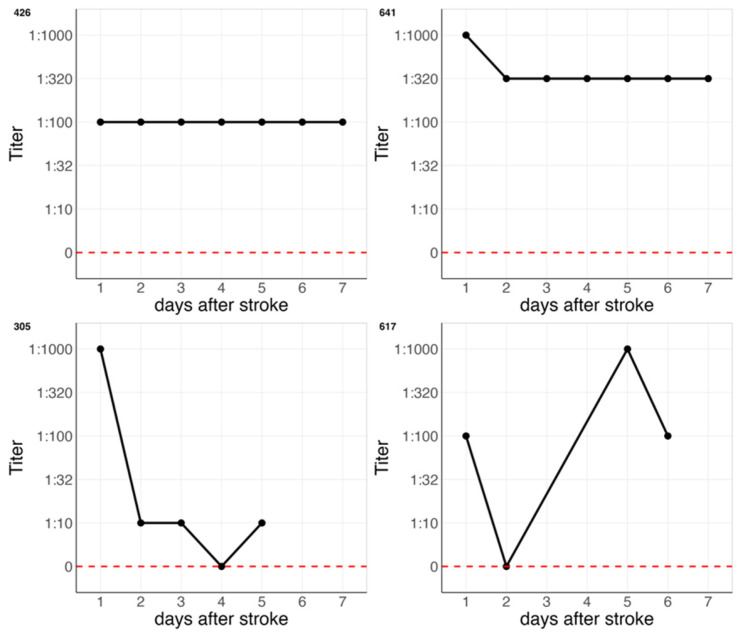
Two representative examples of stable course (**upper row**), 426 and 641), and two representative examples of a fluctuating course (**lower row**), 305 and 617) of NMDAR1-abs titer levels over 7 consecutive measurements in acute stroke. Red dashed line indicates a titer of 0, which equals seronegativity in our study.

Regarding the different Ig-isotypes, all patients who were seropositive for either IgM or IgA at the first measurement, were also seropositive for either IgM or IgA, at the last measurement, respectively. Out of 26 stroke patients with any follow-up measurement, 11 individuals had NMDAR1-abs IgM titers at day one after stroke, and three individuals IgA titers. Titer levels were available in 58 out of 72 (81%) seropositive samples, in total, because in some sera, the volume was too low for titration. Titer levels were foremost stable or with minor variation in these patients; i.e., in 9 out of 11 patients with IgM (82%) and in 2 out of 4 patients with IgA (50%), the titer was stable according to our definition. In total, 8 out of 12 patients (67%) had a stable titer. Examples of a stable titer course are shown in the upper row of Figure 2 (identifier: 426, 641), and examples of a fluctuating titer in the lower row of Figure 2 (identifier: 305, 617). Titer levels showed overall neither an increasing nor a decreasing trend. Despite the small sample size, baseline clinical characteristics did not provide any indication of whether NMDAR1-abs titers were stable, as shown in Supplemental Table S1. Titer levels over all consecutive measurements are displayed for IgM in Supplemental Figure S1 and for IgA in Supplemental Figure S2.

### 3.3. Clinical Outcomes at 90 Days and 6 Months After Stroke

**Figure 3:**
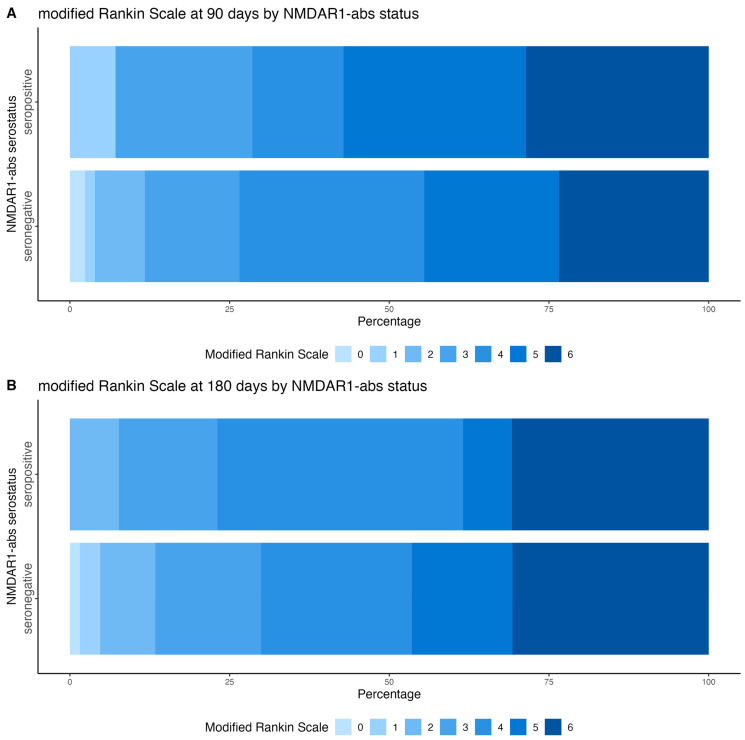
Grotta bars showing percentages of individuals within categories of the modified Rankin Scale 90 (**A**) and 180 (**B**) days after stroke stratified by anti-N-methyl-D-aspartate-receptor antibody serostatus.

We explored clinical outcomes of 171 patients with antibody measurement. From six patients, clinical information was completely missing at day 90 and at 6 months after stroke. At 90 days after stroke, 43 out of 165 patients (21%) had died, of whom four were seropositive (25%) and 30 seronegative (20%). mRS at day 90 was missing in 30 individuals, and we recorded unacceptable mRS (i.e., >4) in 64 (45%) patients, of whom eight were seropositive (57%) and 56 were seronegative (44%). Six months after stroke, no additional seropositive patient had died, however, seven more seronegative patients, rendering a total of 25% of patients who died, were also in the seronegative group. mRS was missing in 32 patients 6 months after stroke. Five out of 13 (38%) seropositive, and 58 out of 126 (46%) seronegative patients had unacceptable mRS. Percentages in each category of the mRS at 90 and at 6 months after stroke, stratified by NMDAR1-abs serostatus, are shown in Figure 3.

## 4. Discussion

NMDAR1-abs of the IgA and IgM isotype were present in 9% of patients with severe acute MCA infarction. NMDAR1-abs serostatus (i.e., seropositive vs. seronegative) was stable over seven consecutive days following stroke. Titer levels for either Ig-isotype remained largely stable.

Stroke is a devastating clinical event, and the risk factors contributing to unfavorable outcomes remain insufficiently understood. Emerging evidence suggests a potential role of NMDAR1 antibodies (NMDAR1-abs) as biomarkers for identifying patients at increased risk, particularly for neuropsychiatric sequelae such as cognitive impairment [10]. In the present study, we were only able to replicate partially the high frequency of serum NMDAR1-abs reported in earlier studies, where prevalence in individuals with various disorders (including stroke) ranged from 10% to 20% [3,4]. In contrast, the observed prevalence in our cohort was slightly lower at 9%. This finding was somewhat unexpected given the advanced age and severity of stroke events in our patient population. An explanation for the discrepancy between the observed prevalence in our study compared to others may reflect random variation due to the relatively small sample size. However, early immunoprecipitation following stroke, i.e., the immediate binding of circulating antibodies to their neural targets, serves as an alternative explanation, as previously proposed [13]. Given (i.) the observed prevalence in our study (slightly lower compared to others) in the context of the timing of antibody measurement and (ii.) the relative stability of titer levels (no increasing trend), we consider it unlikely that the stroke event itself induced NMDAR1-abs formation. Other studies previously indicated, that NMDAR1-abs are not hypermutated, thus likely belonging to the germline antibody repertoire [14].

Previous studies showed that serum NMDAR1 antibodies are predominantly of the IgA and IgM isotypes (>95%). Accordingly, for economic reasons, we limited our measurements to these two isotypes. In the context of stroke, NMDAR1-abs seropositivity was associated with unfavorable outcomes [8]. The primary finding of the present study is, that NMDAR1-abs serostatus remained stable during the acute phase of stroke, supporting potential as a robust biomarker. We observed one exception: one individual was initially seronegative for both isotypes but was measured with a low IgM titer of 1:10 on day two. Given the low titer, this may represent a false-positive result [15]. It is important to note that CBAs are subject to rater interpretation [16], which may account for minor variability. Two seropositive individuals exhibited highly fluctuating titer levels over time, including transient seronegativity, followed by reversion to seropositive status at the final measurement (see both examples in Figure 2). To date, we are unable to attribute these fluctuations or serostatus conversions to any identifiable clinical characteristics. Therefore, the meaning of such fluctuating titers remains unknown.

Our findings are in line with a previous study investigating the stability of NMDAR1-abs serostatus following stroke. A 2016 study suggested that NMDAR1-abs seropositivity may be a relatively stable marker in the acute phase of stroke, although that study measured NMDAR1-abs at three time-points, only. Specifically, NMDAR1-abs values were measured on day one, two, and seven after stroke onset. A uniform but modest decline in titers was reported from day one to two [17]. While a similar trend was observed in some individuals in our study, our data do not uniformly confirm this early decline in titers.

Another study investigated the long-term stability of NMDAR1-abs serostatus. The NMDAR1-abs value was measured shortly after the stroke event and at a follow-up time point between one and three years later. The overall prevalence of seropositivity remained consistent at 20% from baseline to follow-up, although intraindividual fluctuations in titers were noted [18]. Notably, this study reported a higher proportion of IgG-type antibodies compared to other studies [8,17], which may account for an overall increased prevalence. Future research should aim to clarify the intra-individual long-term trajectory of NMDAR1-abs seropositivity, including isotype profiles, titer dynamics, and the clinical relevance of these parameters for outcome prediction. These questions are highly relevant, particularly when considering parallels with other antibody-associated neurological diseases. For instance, aquaporin-4 antibodies are recognized as a causal factor in seropositive Neuromyelitis Optica Spectrum Disorders (NMOSD). However, despite extensive research, no consistent correlation between antibody titers and disease severity or clinical course has been established [19]. Similarly, a clinically meaningful cut-off for NMDAR1-abs seropositivity will likely depend on future evidence regarding their predictive properties for outcomes in stroke and other disorders.

In our study, no substantial differences in mortality or mRS were observed between NMDAR1-abs seropositive and seronegative patients. We attribute this to two main factors: first, the follow-up period may have been too short to capture effects that develop chronically or emerge in the long term after stroke; second, the sample size was likely insufficient to detect effects previously reported in other studies. For instance, although our 90-day data suggested higher mRS scores and increased mortality among seropositive individuals, these differences were no longer apparent at the 6-month follow-up.

Moreover, missing data during follow-up may have introduced bias, further limiting interpretability. Importantly, neuropsychiatric outcomes—particularly cognitive function—were not assessed in this study. This represents a key limitation, as cognitive outcomes should be central to evaluating the potential functional impact of NMDAR1-abs, given the critical role of NMDA receptors in synaptic plasticity and memory processes.

This study has several limitations that primarily stem from the secondary use of data and biospecimens originally collected for a different research objective. The population size was relatively small including 171 individuals with NMDAR1-abs measurements and missing data during follow-up. This may be the key limitation of why a link with clinical outcome could not be established. Nonetheless, a key strength of the study lies in its rigorously standardized biosampling protocol, which enhances the reliability of serological measurements. Despite this, we encountered several challenges, particularly related to biospecimen availability. In many cases, residual serum volumes were insufficient or entirely lacking, which may have introduced bias due to missing data. While statistical modeling of intra-individual longitudinal trajectories would be desirable for stronger conclusions, our data were insufficient to support such analyses. Furthermore, clinical outcome data beyond the acute phase were extremely limited and did not include endpoints most relevant to the evaluation of NMDAR1-abs, such as neuropsychiatric or cognitive outcomes. As a result, this study does not permit conclusive statements regarding the significance of NMDAR1-abs as a biomarker for clinical outcomes. However, it provides valuable insights into the short-term stability of these antibodies, supporting their robustness in acute stroke. However, importantly, the clinical relevance and the validity of positive testing for IgA and IgM NMDAR1-abs require further investigation.

## 5. Conclusions

In summary, NMDAR1-abs serostatus is likely not linked to stroke severity. Any NMDAR1-abs seropositivity can be considered as a robust biomarker in the acute stroke phase, up to seven days after a moderate or severe MCA stroke.

## Figures and Tables

**Table 1:** Baseline characteristics of STRAWINSKI participants.

	STRAWINSKI Total	Missing	NMDAR1-Abs Seronegative	NMDAR1-Abs Seropositive
**Patients** *n* (%)	166 (100)	5 (3)	-	16 (10)
**Age in years** *mean* (SD)	76 (11)		76 (11)	77 (11)
**Female sex** *n* (%)	92 (55)		87 (58)	5 (31)
**NIHSS** *median* (IQR)	15 (12–18)		15 (12–18)	15 (12–16.5)
**Moderate stroke event** NIHSS 9–15 *n* (%)	102 (61)		92 (61)	10 (63)
**Severe stroke event** NIHSS > 15 *n* (%)	64 (39)		58 (39)	6 (38)
**Previous stroke event** *n* (%)	41 (25)	8 (5)	37 (25)	4 (25)
**History of hypertension** *n* (%)	147 (86)		131 (87)	16 (100)
**History of diabetes** *n* (%)	48 (29)	4 (2)	40 (27)	8 (50)
**History of atrial fibrillation** *n* (%)	84 (51)	7 (4)	75 (50)	9 (56)
**Smokers** *n* (%)	23 (14)	25 (15)	21 (14)	2 (12.5)
**Care situation before stroke** *n* (%)				
Independent at home	121 (73)		111 (74)	10 (63)
Home care	25 (15)		24 (16)	1 (6)
Care facility	20 (12)		15 (10)	5 (30)
**TOAST** *n* (%)		2 (1)		
LAA	45 (27)		39 (26)	6 (38)
CE	81 (49)		73 (49)	8 (50)
SVD	5 (3)		5 (3)	0
Undetermined	3 (2)		3 (2)	0
Unknown	30 (18)		28 (19)	2 (13)

STRAWINSKI, Stroke Adverse Outcome is Associated with Nosocomial Infections trial; TOAST, Trial of Org. in acute Stroke Treatment criteria; LAA, large artery artherosclerosis; CE, cardioembolic; SVD, small vessel disease; n, number; SD, standard deviation; IQR, bounderies of the interquartile range; Missing, missing information from total; Charasteristics and column headers are in bold. Descriptive statistics are in italic.

## Data Availability

Data and analytical scripts are available from the corresponding author (pia.sperber@charite.de) upon reasonable request.

## Supplementary Materials

The following supporting information can be downloaded at: https://www.mdpi.com/article/10.3390/diagnostics15243132/s1. Table S1: Characteristics of stroke patients with a stable titer and those with a fluctuating titer. Figure S1: Anti-NMDA-IgM titer levels over up to seven consecutive measurements. Figure S2: Anti-NMDA-IgA titer levels over up to seven consecutive measurements.
