# Supplementary material for: Anti-NMDA-Receptor GluN1 Antibody Serostatus Is Robust in Acute Severe Stroke
Source: Diagnostics (Basel). 2025 Dec 9;15(24):3132. doi: 10.3390/diagnostics15243132 (PMC12731413; doi:10.3390/diagnostics15243132)
Supplement: Supplementary file 1 [file diagnostics-15-03132-s001.zip › diagnostics-3997183-supplementary.pdf]

SUPPLEMENTAL MATERIAL

Anti-NMDA-receptor GluN1 antibody serostatus is robust in acute severe stroke

Pia S. Sperber[1-4], Benjamin Hotter[1,4], Matthias Endres[1,3-5], Harald Prüß[1,6], Andreas Meisel[1,3-5]

[1]Charité-Universitätsmedizin Berlin, corporate member of Freie Universität and Humboldt-Universität zu Berlin, Department of Neurology and Experimental Neurology, Berlin, Germany
[2]Experimental and Clinical Research Center, a cooperation between Max Delbrück Center for Molecular Medicine in the Helmholtz Association and Charité-Universitätsmedizin Berlin, corporate member of Freie Universität Berlin and Humboldt-Universität zu Berlin, Berlin, Germany
[3]Charité – Universitätsmedizin Berlin, corporate member of Freie Universität and Humboldt-Universität zu Berlin, Neuroscience Clinical Research Center, Germany
[4]Germany Center for Cardiovascular Diseases (DZHK), partner site Berlin, Berlin, Germany
[5]Charité – Universitätsmedizin Berlin, corporate member of Freie Universität and Humboldt-Universität zu Berlin, Center for Stroke Research Berlin, Germany
[6]German Center for Neurodegenerative Diseases (DZNE) Berlin, Berlin, Germany

Corresponding author:
Pia Sophie Sperber (MD)
Department of Neurology with Experimental Neurology
pia.sperber@charite.de
ORCID: 0000-0002-9534-2374
Charitéplatz 1, D-10117 Berlin, Germany

Supplemental Table S1: Characteristics of stroke patients with a stable titer and those with a fluctuating titer

Patients with	**Stable titer (N=9)**	**Fluctuating titer (N=4)**
**age** mean (SD)	73.3 (12.5)	80.3 (7.80)
**sex**		
Female	1 (11.1%)	3 (75.0%)
Male	8 (88.9%)	1 (25.0%)
**NIHSS baseline** median [Min, Max]	15.0 [10.0, 32.0]	14.0 [11.0, 20.0]
**mRS baseline** median [Min, Max]	5.00 [2.00, 5.00]	4.50 [4.00, 5.00]
**mRS before stroke** median [Min, Max]	0 [0, 3.00]	0 [0, 3.00]

Patients with	Stable titer (N=9)	Fluctuating titer (N=4)
**previous stroke**		
yes	3 (33.3%)	1 (25.0%)
no	5 (55.6%)	3 (75.0%)
Missing	1 (11.1%)	0 (0%)
**arterial hypertension**		
yes	9 (100%)	4 (100%)
no	0 (0%)	0 (0%)

<u>Supplemental Figure 1: Anti-NMDA-IgM titer levels over up to 7 consecutive measurement</u>

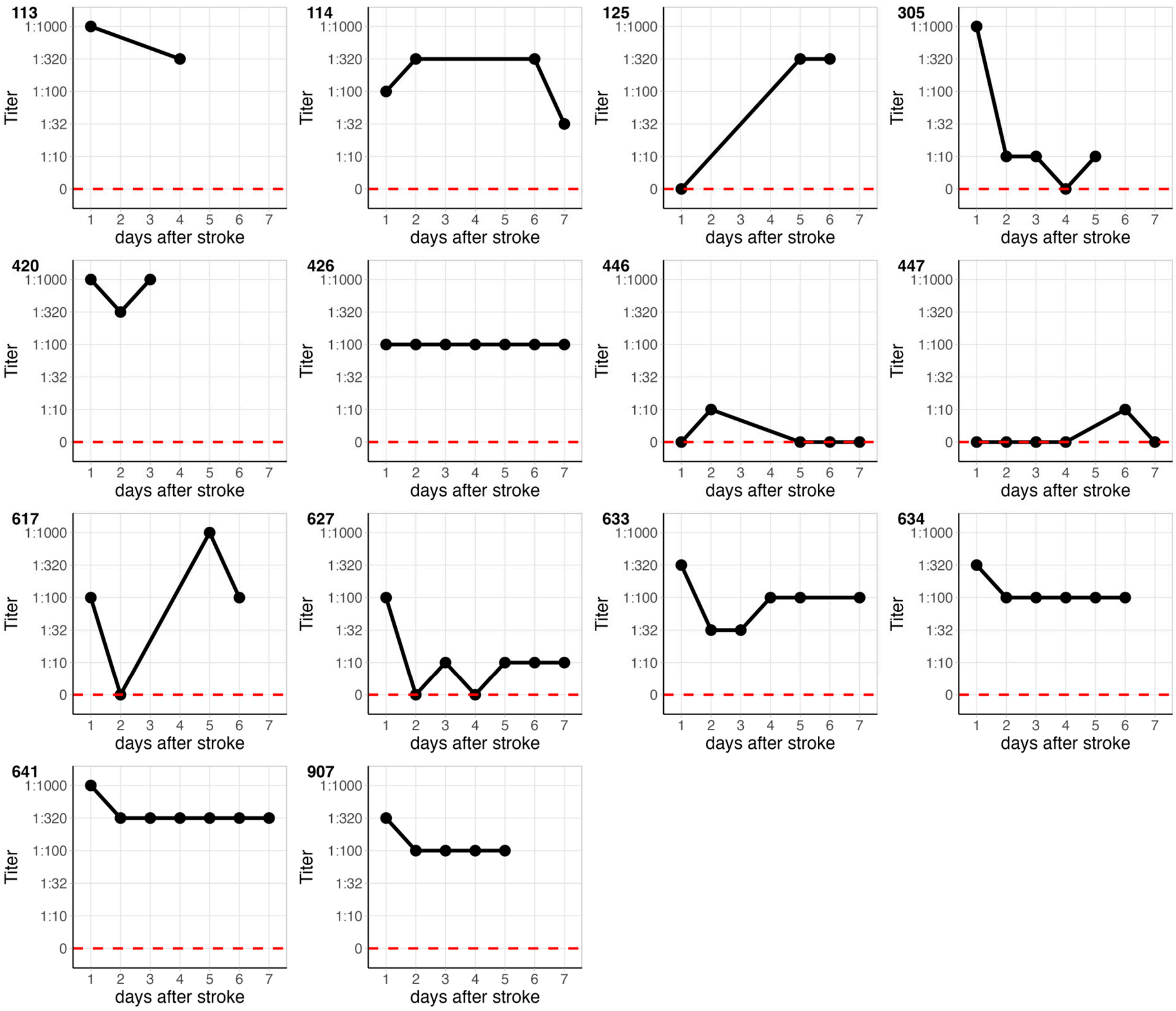

<u>Supplemental Figure 2: Anti-NMDA-IgA titer levels over up to 7 consecutive measurement</u>

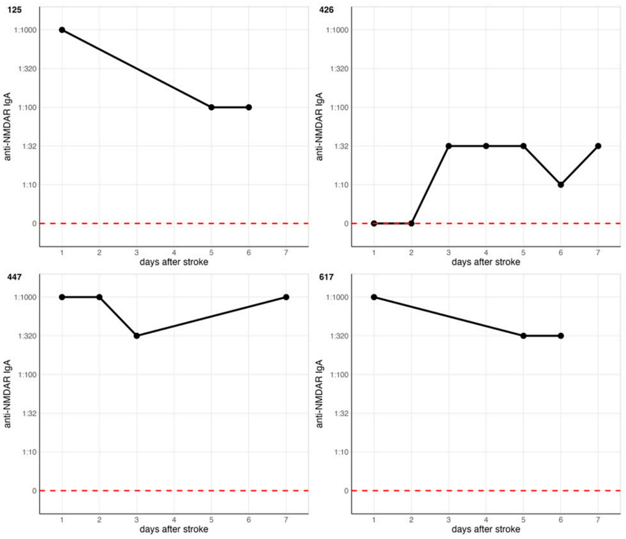

125
426
447
617
anti-NMDAR IgA
1:1000
1:320
1:100
1:32
1:10
0
days after stroke
1
2
3
4
5
6
7
